# Supplementary figures and images for: Late Jurassic teeth of plesiosauroid origin from the Owadów-Brzezinki Lägerstatte, Central Poland
Source: PeerJ. 2023 Jul 14;11:e15628. doi: 10.7717/peerj.15628 (PMC10351514; doi:10.7717/peerj.15628)

## Supplement 2

### Eigenvalues and PCoA results for Plesiosauria

| Axis | Eigenvalue | Percent  |
|------|------------|----------|
| 1    | 0.88572    | 43.153   |
| 2    | 0.30523    | 14.871   |
| 3    | 0.13486    | 6.5703   |
| 4    | 0.092877   | 4.525    |
| 5    | 0.074476   | 3.6285   |
| 6    | 0.052233   | 2.5449   |
| 7    | 0.039343   | 1.9168   |
| 8    | 0.030102   | 1.4666   |
| 9    | 0.024593   | 1.1982   |
| 10   | 0.018007   | 0.8773   |
| 11   | 0.014613   | 0.71196  |
| 12   | 0.011451   | 0.55788  |
| 13   | 0.0085585  | 0.41698  |
| 14   | 0.0052774  | 0.25712  |
| 15   | 0.0042913  | 0.20908  |
| 16   | 0.0032969  | 0.16063  |
| 17   | 0.0023737  | 0.11565  |
| 18   | 0.0018159  | 0.088473 |
| 19   | 0.00095975 | 0.04676  |
| 20   | 0.00049405 | 0.024071 |

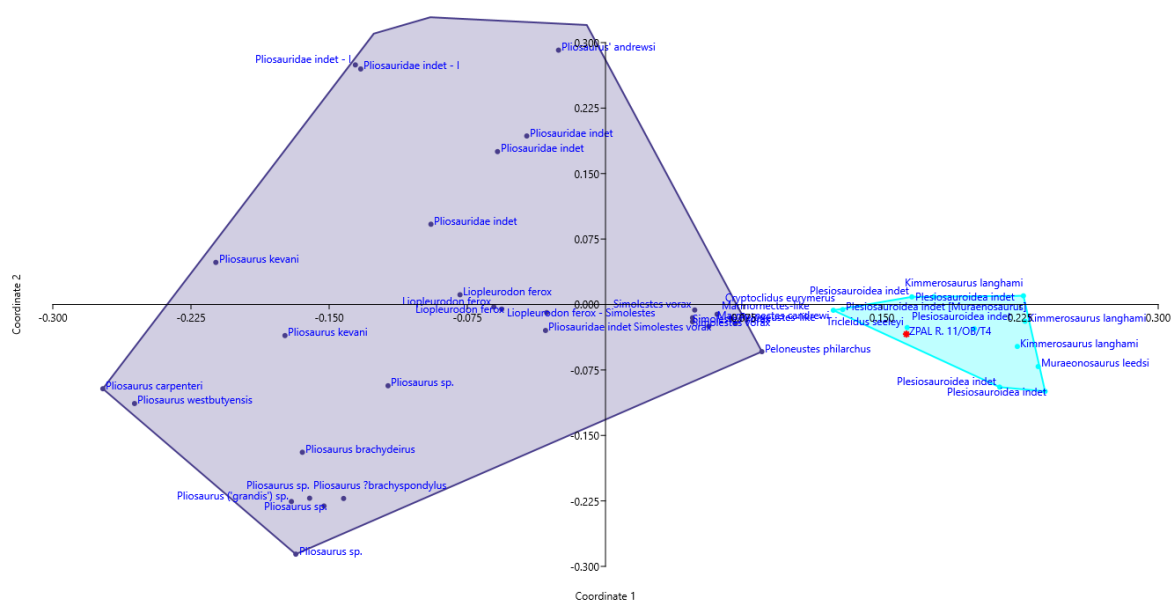

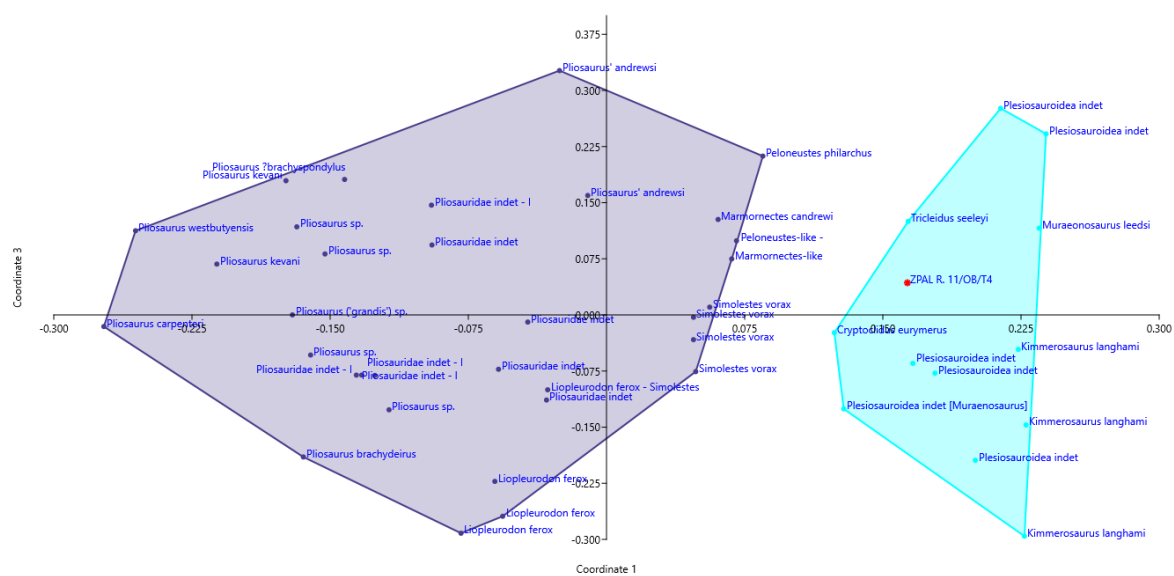

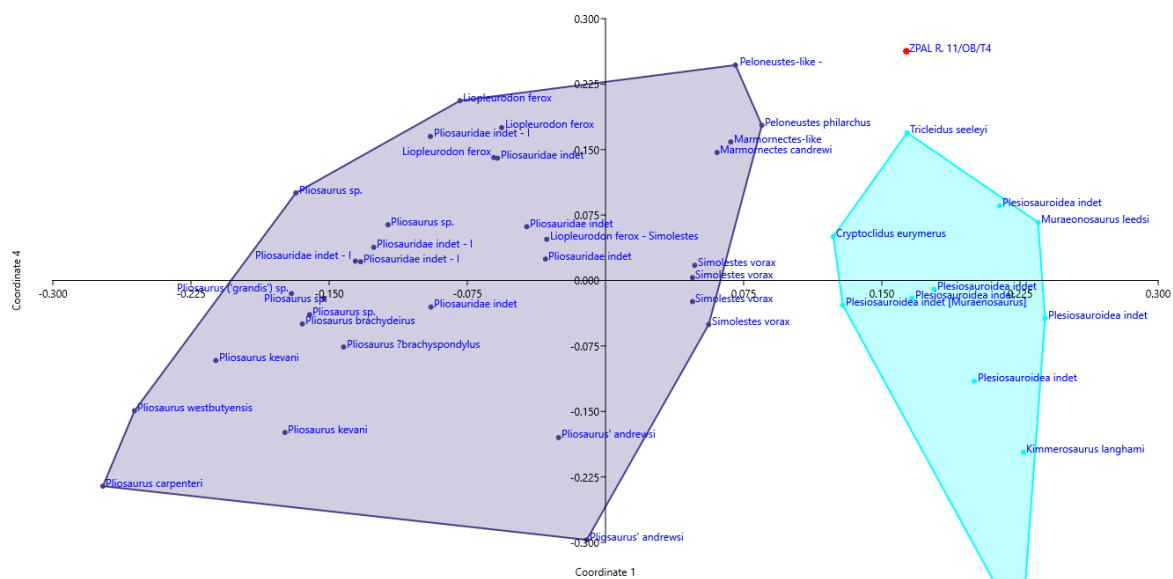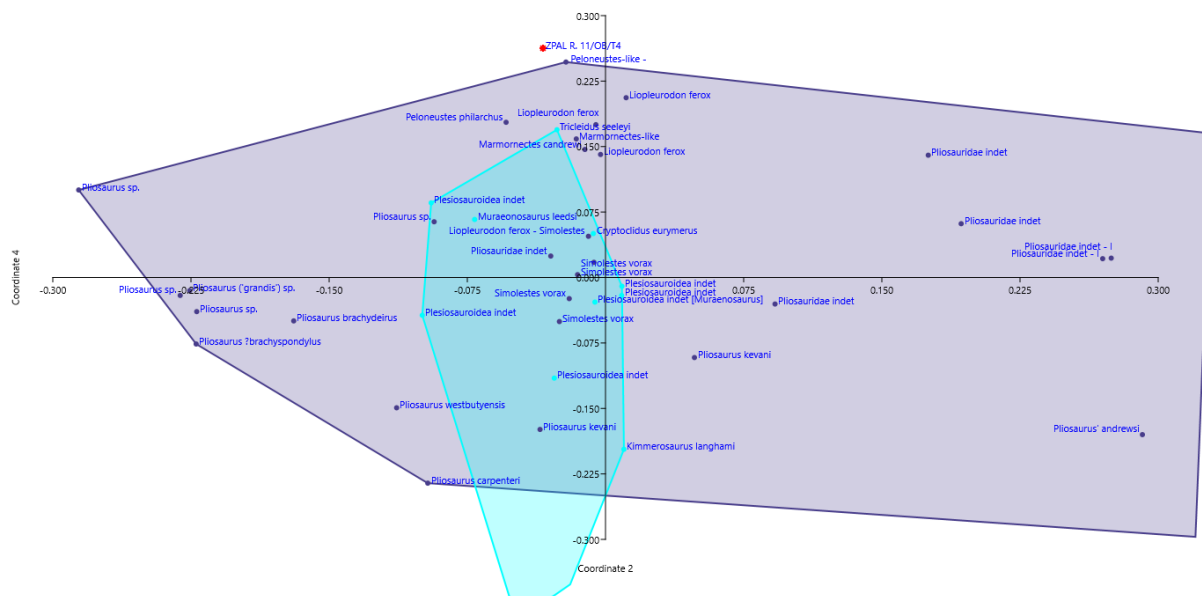

Supplement: Supplemental Information 2 — Results of PCoA from Madzia, Szczygielski & Wolniewicz (2021) modified from Foffa et al. (2018) with data gathered from specimen ZPAL R. 11/OB/T4 compared with Plesiosauria. Eigenvalues for analysis and plots compared against other coordinates. [file peerj-11-15628-s002.pdf]
